# A patient journey map study on time toxicity among young and middle-aged patients with breast cancer

**DOI:** 10.3389/fpubh.2026.1883712

**Published:** 2026-09-01

**Authors:** Lixia Cui, Yuxiang Guan, Xiaoyan Chen, Fenglin Sun, Hao Wu, Yao Wang

**Affiliations:** 1 School of Nursing, Anhui University of Chinese Medicine, Hefei, China; 2 Laboratory of Geriatric Nursing and Health, Anhui University of Traditional Chinese Medicine, Hefei, China; 3 The First Affiliated Hospital of Anhui University of Chinese Medicine, Hefei, China

**Keywords:** breast neoplasms, patient journey mapping, qualitative research, time toxicity, young and middle-aged

## Abstract

**Objective:**

Based on the journey map, this study identified the multidimensional real-world experiences and core pain points of time toxicity among young and middle-aged breast cancer patients throughout the entire disease course, to provide a reference for alleviating patients’ time burden, optimizing the medical experience, and improving their quality of life.

**Methods:**

A descriptive qualitative study was conducted. From August 2025 to February 2026, purposive sampling was used to recruit 19 young and middle-aged patients with breast neoplasms undergoing treatment at a tertiary Class A hospital in Anhui Province. Data from semi-structured in-depth interviews were analyzed using conventional content analysis.

**Results:**

Participants experienced three sequential clinical phases including screening and diagnosis, treatment preparation, and treatment implementation. Their time toxicity experiences were categorized into four dimensions, namely behaviors, emotions, touchpoints and pain points, generating 19 phase-specific subthemes in total. Two cross-cutting core themes running through the full care pathway were further summarized from repeated shared experiences. Based on these findings, a patient journey map of time toxicity was developed for young and middle-aged patients with breast cancer.

**Conclusion:**

The experience of time toxicity in young and middle-aged breast cancer patients is dynamic and phase-specific. It is characterized by passive waiting and information asymmetry during screening; decision-making pressure, role transition, and time conflict during treatment preparation; and cumulative time costs and social role deprivation during treatment implementation. We recommend phased and precise intervention strategies to better balance survival benefits and quality of life in this population.

## Introduction

Breast cancer is the most prevalent malignant tumor among women in China. Compared with Western countries, breast cancer in China demonstrates a notable younger age at onset, with 68.7% of patients aged under 60 years and a relatively high proportion of young and middle-aged individuals (1). This population is typically at a critical life stage involving heavy family responsibilities and career development, including supporting older adult parents, raising children, maintaining family life, and pursuing occupational advancement (2).

Treatment for breast cancer is a long-term process that requires frequent hospital visits, with substantial time devoted to traveling, waiting, receiving treatment, and recovering from adverse reactions. For young and middle-aged patients with limited time resources, such demands easily lead to a considerable time burden, which directly impairs family functioning, career development, and treatment adherence. The term “time toxicity” was first coined by Daily (3) to describe unrecognized time-related burdens among cancer patients receiving treatment. Later, Nwozichi et al. (4) standardized this construct via systematic concept analysis, defining time toxicity as the cumulative time burden associated with treatment-related activities imposed by complex and prolonged therapeutic regimens. This burden disrupts daily life, induces emotional distress, and adversely affects quality of life and treatment compliance. Evidence suggests that the excessive time costs of cancer treatment may even attenuate the survival benefits of therapy (5), reducing time available for family and personal activities and further impairing quality of life. Patient journey mapping integrates fragmented care experiences into a chronological narrative, providing a holistic understanding of multi-dimensional interactions and informing service improvements. This approach has shown clear value in chronic disease management and health service optimization (6).

Current research on treatment-related toxicity in breast cancer has focused mainly on physical toxicity, while investigations of time toxicity remain limited. Most existing studies are quantitative and lack an in-depth exploration of patients’ subjective experiences. Therefore, this study used patient journey mapping to illustrate the full trajectory of young and middle-aged breast cancer patients, identify core experiences, key triggers, and pain points of time toxicity, and provide evidence for the development of targeted clinical interventions.

## Methods

### Sample and recruitment

Using purposive sampling and maximum difference sampling, the study recruited middle-aged and young women with breast cancer who were receiving treatment at the Oncology Department and Breast and Gynecologic Oncology Department of a Grade A Level III hospital in Anhui Province between August 2025 and February 2026. Inclusion criteria: ① Histopathologically confirmed diagnosis of breast cancer, with diagnostic criteria conforming to the *Chinese Anti-Cancer Association Guidelines and Standards for the Diagnosis and Treatment of Breast Cancer (2024 Edition)* (7); ② According to the World Health Organization (WHO) age grouping criteria, the middle-aged and young adult group is defined as 18 ~ 59 years old (8); ③ Patients with literacy skills and no cognitive impairment; ④ Patients aware of their diagnosis;⑤ No predefined cut-off time point was set for treatment progress; patients at any stage of disease care (screening and diagnosis, preoperative preparation, postoperative systemic therapy, recurrent multi-line treatment) were eligible for enrollment. Exclusion criteria: ① Patients who interrupted treatment during the study period due to disease progression, transfer to another hospital, or other reasons, and were unable to provide complete information regarding their experience from diagnosis to the treatment phase. Maximum difference sampling was conducted using age, disease stage, employment status, place of residence, and current treatment phase as screening dimensions, to recruit participants with diverse treatment trajectories and improve the heterogeneity of interview data. The sample size was determined according to the principle of inductive thematic saturation (9). During the concurrent process of data collection and preliminary analysis, thematic saturation was deemed preliminarily achieved when the analysis of interview data from three consecutive participants failed to yield any new themes or subthemes. To enhance the robustness of the conclusions, two additional interviews were conducted on this basis. Recruitment was halted after confirming that no new information emerged. A total of 19 young and middle-aged female breast cancer patients were interviewed. This study was approved by the hospital ethics Committee (approval no.: 2024AH-89; approval date: September 18, 2024) and adhered to the Consolidated Criteria for Reporting Qualitative Research (COREQ). Written informed consent was obtained from all participants before enrollment. All interviews were audio-recorded, anonymized, and transcribed verbatim.

### Questionnaire development

Based on relevant studies on the experience of time toxicity and journey maps among patients with malignant tumors (10, 11), a preliminary draft of the interview guide was developed. Medical and nursing experts (all holding associate senior or higher professional titles) with ≥10 years of clinical or research experience in the field of malignant tumors, as well as researchers with extensive experience in qualitative research, were invited to form an expert panel to revise the interview guide. Prior to the study’s commencement, two middle-aged and young breast cancer patients who met the inclusion and exclusion criteria were selected for pilot interviews (not included in the formal study sample). Based on feedback from the participants and guidance and suggestions from clinical experts, the research team held discussions to finalize the interview guide. The interviewer established rapport by asking, “Would you be willing to share your experiences with your illness and treatment with me?” The subsequent interview guide is as follows: ① At which stage or aspect of treatment did you feel the greatest time pressure? ② How did the emotional impact and practical burden of these treatment-related time commitments (e.g., commuting, waiting, the treatment itself) change as the treatment progressed? ③ How did your treatment schedule affect your studies, work, or family responsibilities? ④ How did you adjust your life to cope with these time pressures? What strategies were most effective? ⑤ What is your biggest challenge regarding time management at present? Are there any other related feelings or thoughts you’d like to share regarding the time commitments associated with treatment?

### Data collection

All interviewers received standardized training in qualitative research and were nursing researchers without clinical practice experience in breast oncology, which effectively reduced inherent *a priori* biases derived from clinical practice. Nevertheless, individual characteristics including researchers’ disciplinary background, age and gender could subtly shape interview questioning orientation and data interpretation outcomes. Standardized interview protocols were developed for sensitive cancer-related interview topics to mitigate such potential confounding biases.

Prior to formal interviews, researchers fully explained the study purpose to subjects and obtained written informed consent, clarifying privacy protection provisions including audio recording confidentiality, subject anonymization, and exclusive academic use of research data. A 5 ~ 10 min informal conversation was arranged at the start of each interview, covering daily work and family care to build rapport and narrow hierarchical power gaps between researchers and patients. All interviews were conducted in quiet private ward compartments, with audio recording permitted in advance by all subjects. The interview schedule could be flexibly adjusted with rest intervals according to subjects’ physical and mental conditions. Researchers maintained empathic listening throughout the process, provided timely emotional relief for negative feelings, and allowed subjects to voluntarily avoid distressing sensitive topics to fully guarantee their right of autonomous expression. Subjects’ nonverbal behaviors and emotional fluctuations were objectively documented without leading questions.

Data saturation was achieved after recruiting 17 subjects, and two additional interviews yielded no emerging new themes. Each interview lasted 30 ~ 40 min. Reflective field notes were completed within 24 h after each interview to objectively record on-site details, subjects’ emotional states and researchers’ subjective presuppositions. Biweekly team seminars were held during coding to identify and revise subjective interpretive biases. All reflective records were archived to form complete audit trails. After transcription, all verbatim manuscripts were returned to subjects for member checking and validation.

### Data organization and analysis

Audio recordings were transcribed verbatim within 24 h of each interview. Two researchers independently verified and cross-checked transcripts against original recordings and field notes to ensure accuracy. All interviews were labeled P1–P19 and managed using NVivo 20.0 software. Given the limited literature on time toxicity in breast cancer, conventional content analysis was used for data analysis. The analytical steps were as follows: ① Two researchers repeatedly read all transcripts to achieve immersion; ② Line-by-line open coding was performed to extract meaningful units; ③ Similar codes were categorized, compared, and merged into subthemes and themes; ④ Final themes and subthemes were defined, and representative quotations were selected (12).

### Rigor

We applied strategies to ensure the rigor and trustworthiness of this study.

1. Credibility: before interviews, clinical and contextual information was reviewed to tailor questions and reduce recall bias. Probing and clarification were used during interviews, and non-verbal cues were noted. All transcripts were verified by participants through member checking within 24 h.
2. Dependability: two researchers coded data independently and cross-checked themes; any discrepancies were resolved via group discussion.
3. Confirmability: peer debriefing was introduced as an additional safeguard. An external specialist with professional qualitative research training reviewed the complete coding logic and thematic framework to conduct third-party validation, further ensuring neutral analytical outcomes.

## Results

A total of 19 young and middle-aged breast cancer patients were enrolled, and all participants received systemic anti-tumor therapy after surgery. The distribution of systemic treatment was neoadjuvant chemotherapy (*n* = 2, 10.53%), postoperative chemotherapy (*n* = 10, 52.63%), postoperative targeted therapy (*n* = 4, 21.05%), and recurrent/multiple-line therapy (*n* = 3, 15.79%). No patients received radiotherapy because our hospital has no radiotherapy equipment; all patients will transfer to external hospitals for radiotherapy after finishing all cycles of systemic treatment.

This study recruited 19 breast cancer patients aged 19 ~ 55 years (mean 38.95 ± 9.12 years). Most participants were married with one child; 36.84% took medical leave and 21.05% were unemployed, with 57.89% being local residents. Stage II was the predominant disease stage, and the median disease duration was 7 months. Postoperative chemotherapy was the main treatment. Demographic, clinical and surgical subtype characteristics are shown in Table 1.

**Table 1:** Participant characteristics (*n* = 19).

Variable	Category	*n*(%)
Age (years)	Mean ± SD	38.95 ± 9.12
Marital status	Married	16 (84.21)
	Unmarried	2 (10.53)
	Divorced	1 (5.26)
Number of children	0	3 (15.79)
	1	14 (73.68)
	2	2 (10.53)
Employment status	Medical leave	8 (42.11)
	Unemployed	4 (21.05)
	Full-time	3 (15.79)
	Resigned	2 (10.53)
	Part-time	2 (10.53)
Residence	Local	11 (57.89)
	Non-local	8 (42.11)
Disease stage	IA	1 (5.26)
	IIA/IIB	12 (63.16)
	IIIA	2 (10.53)
	IV	4 (21.05)
Disease duration (months)	Median (range)	7 (4–53)
Current treatment phase	Postoperative chemotherapy	10 (52.63)
	Postoperative targeted therapy	4 (21.05)
	Neoadjuvant chemotherapy	2 (10.53)
	Recurrent/multiple-line therapy	3 (15.79)
Surgical subtype (new)	Breast-conserving surgery (BCS)	7 (36.84)
	Modified radical mastectomy (MRM)	12 (63.16)

**Figure 1:**
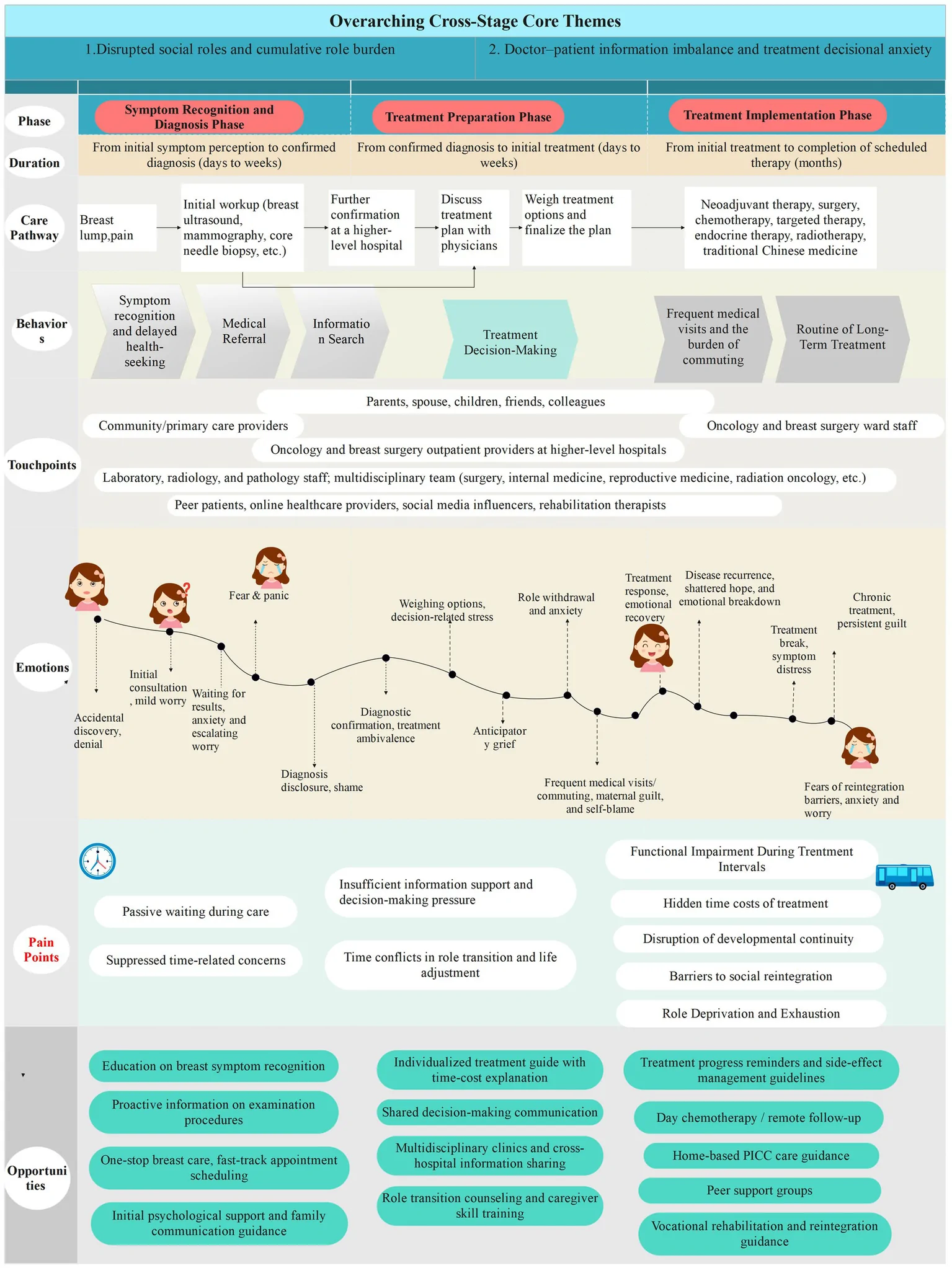
Journey map of time toxicity in young and middle-aged breast cancer patients.

A patient journey map was constructed in this study (Figure 1). The map consisted of a horizontal timeline dimension and a vertical behavioral dimension. The horizontal timeline divided the entire care process into three phases: Symptom Recognition and Diagnosis Phase, treatment preparation, and treatment implementation; the vertical dimension covered four categories: behavioral activities, touchpoints, emotional experiences, and pain points. Touchpoints represent key interaction nodes between patients and healthcare systems throughout care, identified through iterative reading and thematic coding of interview transcripts. Distinct from experiential themes including behavioral activities, emotional experiences and pain points, touchpoints provide contextual background for these experiences. Hence, they are visualized in the patient care journey framework rather than discussed in an independent subsection in the Results and support the analysis of time toxicity across the other three dimensions. Based on this analytical framework, 19 phase-specific subthemes were extracted, which were further sorted into 21 experiential entries visualized in Figure 1. The Opportunities presented in Figure 1 were synthesised by the research team based on participants’ narratives of time-related burdens and the pain points identified in this study. These interpretations were generated after thematic analysis and incorporated into the patient journey map in the final drafting phase.

With the patient journey map, this study identified three interrelated dimensions of time toxicity: core lived experiences, key triggering factors, and pain points.

1. Core lived experiences: holistic subjective psychological perceptions induced by persistent medical time consumption, such as role deprivation, anticipatory grief and social isolation, representing the internal psychological impacts of time toxicity.
2. Key triggers: objective time-consuming medical and life events (e.g., repeated hospital visits, cross-hospital consultation, prolonged waiting) that act as the external sources of time burden.
3. Pain points: tangible adverse outcomes stemming from sustained time loss, including passive waiting, doctor-patient information asymmetry and disrupted personal development trajectories.

Triggers are objective events, pain points are practical negative results, and core experiences are inner psychological responses. Together they form a causal chain: objective triggers consume patients’ time and sequentially create pain points and negative subjective experiences, fully explaining the complete connotation of time toxicity in young and middle-aged breast cancer patients. Stage-specific variations and logical relationships of the three dimensions are illustrated in Figure 1.

Surgical care brings distinct time burdens throughout patients’ treatment journey, all of which are covered in the 21 extracted themes. Some patients visited multiple hospitals for second medical opinions, causing extra travel and consultation time. During treatment preparation, patients spent much time comparing breast-conserving surgery and mastectomy, leading to prolonged decision-making. Postoperatively, long-term home drainage catheter and wound care plus regular follow-ups occupied plenty of daily time and disrupted their work and family life.

### Symptom recognition and diagnosis phase

#### Behavior

##### Symptom recognition and delayed health action

Most participants ignored early breast abnormalities and delayed professional medical visits. This delay created invisible time toxicity. Extended self-observation pushed back formal diagnosis and lengthened the whole care cycle. Three common reasons drove this behavior: misjudging lumps as benign hyperplasia, heavy work and family duties, and limited breast health knowledge. All these factors produced unmeasured hidden time loss before formal treatment began. *P8 “At first, I felt a small lump and thought it was just common breast hyperplasia. I was busy with work and had a lot going on at home, so I figured I’d deal with it once things calmed down”*.

##### Medical referral

Many participants visited multiple hospitals to secure clear diagnoses and better medical resources. Repeated travel, repeated registration and long waiting times all added to time toxicity. Participants distrusted results from primary care facilities. They travelled to higher-level hospitals for second opinions or biopsies unavailable locally, which took up large amounts of personal free time in early diagnosis. *P13 “I was initially examined at the county hospital, but I wasn’t entirely reassured, so I asked around and made an appointment at a major provincial hospital to get a second opinion”*.

##### Information search

After spotting abnormal signs, participants and their families collected disease and treatment information from various sources. Information searching itself consumed spare time. Conflicting online medical content triggered anxiety and slowed treatment decisions, which jointly worsened time toxicity. Participants spent extra free time sorting inconsistent online content without professional clinical guidance. *P11 “The more I searched, the more scared I got. The information online is so mixed—some say it’s fine in the early stages and surgery will fix it, while others say the recurrence rate is high. Every sentence I read made my heart sink. I felt lost, not knowing which to believe, and even thought it might be better not to search at all”*.

#### Emotions

##### Stress-induced negative emotions

Participants lived in fear and distress from discovering symptoms until confirmed diagnosis. Constant worry reduced efficiency at work and housework, which indirectly wasted extra time. Some participants hid their illness due to embarrassment over breast conditions. They processed all information alone and endured longer mental stress without family support. *P13 “When I found out it was cancer, my legs went weak, and I couldn’t stop the tears from falling. I was terrified”*.

#### Pain point

##### Passive waiting

All participants reported unavoidable waiting for blood tests, imaging scans and pathology reports. Unplanned waiting formed the core visible part of time toxicity. Same-day ultrasound appointments were often unavailable, and long waits for pathology results forced extra hospital trips and created large amounts of unproductive empty time. *P10 “Those few days waiting for the pathology report were pure torture. I didn’t know if they’d call, so I was just hoping for some definite news. I had no idea what to expect—whether the results were good or bad”*.

##### Suppressed patient voices

Objective information asymmetry and power imbalance between clinicians and patients throughout treatment prevent patients from fully voicing their doubts and concerns, resulting in suppressed patient voices. On one hand, patients tend to hesitate to raise questions, subjectively believing such inquiries are meaningless and that all clinical arrangements are medically mandatory. On the other hand, patients worry that challenging frequent examinations or treatment plans may leave clinicians with the impression of poor treatment compliance, so they choose to withhold their true thoughts. *P2 “I didn’t dare ask; asking was pointless. They’d just say, ‘This is all necessary.’” P10 “If you tell the doctor the tests are too frequent, they might think you’re questioning their arrangements”*.

### Treatment preparation phase

#### Behavior

##### Treatment decision-making

Treatment selection directly shapes patients’ overall time burden, and participants exhibited two distinct attitudes toward the timing of treatment initiation. Some hoped to accelerate and condense treatment schedules to accommodate career and personal plans. Others delayed treatment due to competing family and work commitments, which extended total care duration and created additional time costs. Patients also varied substantially in their level of involvement during treatment discussions. Many weighed alternative regimens by integrating clinical advice, anticipated therapeutic benefits, and personal time constraints. Several participants balanced treatment options by considering medical recommendations, expected efficacy, and time-related burdens. *P6 “My plan is neoadjuvant therapy followed by surgery. The doctor said it could shrink the tumor and give me a chance to preserve my breast without an implant. Also, since I’m graduating and looking for a job, I’m hoping to shorten the interval between targeted therapies”*. *“P8 “I just wanted a simpler treatment plan; I thought that if I had a total mastectomy, I wouldn’t need radiation therapy”*. Conversely, some patients passively accepted clinician-recommended regimens without raising scheduling concerns aligned with daily routines. This lack of proactive communication often led to mismatches between treatment timetables and personal responsibilities, requiring repeated hospital visits and protocol revisions that amplified time strain. Variations in treatment timing and decision engagement exacerbate time toxicity and constitute a key component of this study’s central theme.

#### Emotion

##### Decision-making conflict

Multiple life demands, conflicting online medical information and uncertain prognosis created inner struggles for participants. Hesitation delayed final plan confirmation and lengthened preparation time, producing psychological time toxicity. Young and middle-aged participants feared wrong treatment choices would harm their prognosis and become a burden to their families. They stayed stuck in indecision before signing treatment consent forms. *P11 “I really do not know how to choose. There are all kinds of opinions online. I’m afraid that making the wrong choice will affect the outcome. I’m panicked and torn, and just cannot make up my mind”*.

##### Anticipatory grief

Driven by the lengthy, staged time costs of sequential diagnosis and treatment across the temporal continuum of care, participants developed anticipatory grief, which acts as a typical psychological manifestation of time toxicity, when they predicted treatment would damage their social roles, disrupt daily routines and bring uncertain prognosis. They felt sadness and helplessness well before treatment started. Such negative expectations disturbed daily work schedules and pushed participants to make extra hospital trips for consultation before formal therapy, creating hidden time loss. Worries about chemotherapy side effects further aggravated psychological distress linked to this cumulative time burden. *P7 “I have not even officially started long-term treatment yet, but I’ve already made several trips to the hospital. Plus, since chemotherapy requires hospitalization, there’s no one to look after the kids at home, and my husband has to take frequent time off work—it really weighs on my mind”*.

#### Pain point

##### Insufficient information support and decision-making pressure

More than half of participants could not get systematic, complete treatment-related information. This created heavy decision pressure and prolonged time spent comparing different regimens. Participants dared not ask doctors about long-term impacts on work and daily life. Clinicians only offered short time windows to finalise surgical and systemic treatment plans. Participants rushed to compare multiple schemes within tight deadlines and faced heavier time-related mental strain. *P8 “I’d get the words to my lips but swallow them back. When I asked, they just told me not to worry and to follow the treatment plan”*.

##### Time conflicts between role transitions and daily life

After diagnosis, patients must rapidly shift from their routine social roles—such as workplace employees and family caregivers—to the role of a patient requiring repeated hospital visits and prolonged treatment, which triggers severe time conflicts arising from overlapping social responsibilities. Prior to illness, participants bore long-term duties including elder care, childcare, and full-time work. Regular treatment and hospitalization occupy large blocks of time, leaving no one available to take over daily household matters. *P1 “I have to ask my boss for time off while also arranging for my child’s pickup and care—so many things all piling up at once”*. *P18 “I’ve always taken care of my older adult parents and child at home. Suddenly, I had to be hospitalized for treatment, and I didn’t even have time to find someone to help. My parents are getting older, and my husband is busy with work and has no time, so I just had to make do with a temporary arrangement”*.

### Treatment implementation phase

#### Behavior

##### Frequent medical visits and the burden of commuting

Regular chemotherapy, targeted therapy, PICC maintenance and blood tests required repeated hospital attendance. Cross-hospital treatment, long registration queues and poor appointment coordination created continuous travel burden throughout long-term therapy and occupied participants’ daily available time. *P17 “Chemotherapy, targeted therapy, and all the nursing follow-ups—I’m always running to the hospital”*.

##### Routine of long-term treatment

Treatment and follow-up visits became fixed daily arrangements. Repeated medical schedules took time originally reserved for work, study and social interaction, forming a steady source of time loss in this phase. *P5 “Going to the hospital once every 21 days is like clocking in for work—it’s become a habit”. P14 “Taking medication and maintaining the catheter—I don’t even have to think about it anymore; it’s just like eating”*.

#### Emotions

##### Guilt and self-blame regarding motherhood

Treatment consumed the majority of participants’ time and energy, preventing them from fulfilling their childcare responsibilities as usual. Long-term repeated hospital visits occupied patients’ daily lives and elicited persistent guilt and shame, which continuously disturbed their emotional state during rest and recovery. The inability to strike a balance between regular medical attendance and family care further aggravated cumulative time burdens; such enduring negative emotions constitute distinct emotional manifestations of time toxicity within the temporal dimension of disease management. *P14 “In a situation like mine, I should be the one stepping up to handle things, but because of this illness, I can’t do anything, and my family has to revolve around me and worry about me every day”*.

##### A cycle of hope and disappointment

Brief relief from symptoms brought short-lived hope, while relapse or repeated discomfort triggered emotional breakdowns. Unstable mood disrupted regular daily schedules and created useless repeated mental stress between treatment cycles. *P2 “My mental defenses have completely collapsed. Going to the hospital time and time again feels like seeing a glimmer of hope, and then suddenly, it’s like getting slapped in the face”*.

##### Sense of social isolation

Chronic cyclic treatment persistently depletes participants’ spare time and disrupts their regular social rhythms across the full temporal course of care. Resultant physical debilitation and altered appearance diminished their willingness to engage in social interaction, leading them to actively withdraw from group activities and lose available leisure and communication opportunities. This state of social isolation acts as a prominent psychological outcome and latent derivative of time toxicity throughout late-stage treatment. *P3 “I have time to spend with my child now. I used to love shopping and hanging out with friends, but now I don’t even want to go out—I just can’t muster the energy”*.

#### Pain point

##### Functional impairment during treatment intervals

Repeated multi-cycle chemotherapy triggers persistent adverse physical reactions including fatigue, limb numbness and generalized bone pain. These symptoms restrict participants’ capacity for work, study and household chores throughout the recovery phase, creating unoccupied idle hours and delaying personal and family responsibilities. Such physical limitations consume substantial post-treatment recovery time and constitute tangible physical manifestations of time toxicity within the temporal continuum of oncological care. *P1 “My hands and feet went numb; I couldn’t do anything normally. It lasted for about three days—I really couldn’t stand it”*.

##### Hidden time costs

Hidden time loss covered waiting for appointments, cross-hospital coordination, medical communication, side effect recovery and inefficient clinical workflows. These invisible time losses squeezed time for careers, study and normal daily life and formed a core hidden dimension of time toxicity. Incomplete guidance on follow-up maintenance often led to blind, wasted hospital trips.

*P7 “When I was discharged, they only mentioned that I needed PICC maintenance. They didn’t explain the process, where to go, or that I needed to make an appointment in advance. It wasn’t until I got home that I realized I had no idea what to do, and I ended up making several wasted trips to the hospital”*.

##### Disruption of developmental continuity

Due to prolonged treatment, some respondents were forced to interrupt their original life trajectories. *P6 “My graduation and career plans were completely disrupted. I’m no longer able to run around and stay busy like before; I can only recuperate slowly at home. The rhythm of my life and studies has been completely disrupted”*. *P14 “For more than half a year, my life revolved entirely around the hospital and treatment, and I had to put my work on hold”*.

##### Barriers to social reintegration

Physical limits, changed appearance and reduced vitality blocked participants’ return to social life after treatment. Fear of strange stares kept them away from group events and permanently cut down their personal and social time. *P14 “Since my relapse, I have no strength, and everything is a struggle. On top of that, my appearance has changed, and I no longer have the drive I used to have”*.

##### Role deprivation and exhaustion

Continuous treatment harmed physical function and stripped participants of core work and caregiving roles. Severe physical and mental tiredness followed. Loss of social function stopped participants from splitting energy between hospital visits and family duties. This represented the most serious role-related time burden across all phases. *P2 “My condition relapsed while I was breastfeeding. I had to undergo treatment, couldn’t take care of my child, and couldn’t go to work. I had to handle everything myself—going to the hospital and managing everything on my own. It’s definitely different from others”*. *P14 “I can’t take care of my parents now, nor can I spend enough time with my child. I’m exhausted, and I feel incredibly guilty”*.

The three preceding sections outline behaviors, emotions and pain points specific to each clinical stage. After examining recurring patterns across the full treatment journey, we identified two overarching core themes that cut across all phases. While the previously reported experiences only appear in discrete stages, these two themes run through screening, preparation and treatment implementation alike and constitute the shared core threads behind all time toxicity experiences.

##### Disrupted social roles and cumulative role burden

Recurring hospital visits and sustained treatment undermine patients’ occupational and family care roles. Related stress intensifies along treatment and presents stage-specific features.

Unexplained physical symptoms consume time and energy during screening and diagnosis, triggering mild role impairment.

*P6 “I should have been busy finishing experiments, writing my thesis, and preparing for fall recruitment to find a job. But after the diagnosis, I had to undergo chemotherapy, and my body couldn’t handle it. I had to ask my junior lab mates to help with the experiments, and I couldn’t participate in the fall recruitment. I even had to put aside normal schoolwork and job hunting”*.

To complete examinations and regimen selection in the preparation phase, participants transferred housework, childcare and work duties to relatives. Temporary withdrawal from routine roles induced strong guilt.

*P9 “After the diagnosis, I quit my job and left household chores and childcare behind; I felt guilty about it”*.

Persistent chemotherapy side effects and repeated hospital visits lead to permanent loss of social function in the implementation phase, accompanied by severe physical and mental fatigue and persistent self-blame.

*P14 “Since my relapse, I have no strength, and everything is a struggle. On top of that, my appearance has changed, and I no longer have the drive I used to have”*.

##### Doctor–patient information imbalance and treatment decisional anxiety

Doctor–patient information asymmetry persists throughout all care stages, with unique anxiety triggers at each phase.

Limited disease information fuels anxiety during screening and diagnosis. Uncertain waiting for test results leaves patients vulnerable to panic and helplessness.

*P3 “From the moment they found something abnormal, I was just waiting for the diagnosis—no updates at all in between. I couldn’t focus at work those days; I was constantly checking my phone for news, feeling panicked, with my mind filled with negative thoughts”*.

Participants sourced disease information from diverse channels during treatment preparation. Contradictory online medical content amplified internal confusion. Narrow time windows to confirm regimens further elevated psychological strain.

*P4 “I looked up a lot of information online about neoadjuvant therapy, but the more I read, the more anxious I became—the accounts were all different. Later, when I asked the doctor, he told me not to read random things and just follow his plan, but I still felt uncertain”*.

Insufficient guidance on catheter maintenance, side effect management and follow-up schedules generates redundant hospital visits and prolonged mental distress during treatment implementation. All information-related negative emotions stem fundamentally from doctor–patient information asymmetry.

## Discussion

### Optimizing medical treatment processes and individualized diagnosis and treatment regimens

This study demonstrated that time toxicity among young and middle-aged breast cancer patients exhibited phase-specific characteristics. Passive waiting was a prominent pain point during the screening and diagnosis phase, whereas frequent hospital visits constituted the primary challenge during the treatment implementation phase. Patients required regular attendance for chemotherapy, targeted therapy, PICC maintenance, and follow-up examinations. Those receiving cross-hospital care also encountered difficulties with registration queuing and poor time coordination. Consistent with participants’ shared subjective narratives recorded in our Results, repeated hospital commutes and unavoidable waiting procedures were widely perceived as prominent sources of time burden, consistent with the findings of Jing et al. (11).

Compared with older adult patients, young and middle-aged individuals are in a critical life stage involving substantial family care obligations and career development. Treatment-related time expenditures, including waiting and traveling, directly translate into opportunity costs for fulfilling family and occupational roles. Among the 19 participants in this study, 16 were married and a total of 16 had at least one child; only 5 maintained full-time or part-time employment during treatment. Passive waiting during screening not only induced anxiety but also encroached on the time allocated to childcare and work. The 8 non-local patients receiving cross-hospital care incurred additional hidden time costs related to long-distance transportation, accommodation, and inter-hospital appointment coordination. Furthermore, a lack of mutual recognition of test results between institutions frequently led to redundant examinations and prolonged procedures. These factors contributed to a significantly higher time burden among non-local patients, representing a unique feature of time toxicity in this population.

Drawing on the cross-hospital consultation and redundant examination burdens identified across all three treatment phases in our Results, we tentatively propose developing a one-stop multidisciplinary breast care system integrating screening, assessment, diagnosis and centralized appointment management for future clinical exploration. As reported by Zhang et al. (13), this integrated model may help cut inter-department travel and waiting time and ease time-related burdens; however, its actual efficacy in mitigating time toxicity among local breast cancer patients still requires targeted empirical verification. Meanwhile, intelligent triage systems, dedicated fast-track pathways, and improved cross-hospital information sharing can further streamline care processes and reduce redundant tests (14). Fast-track clinical infusion pathways can effectively optimize medical procedures, cut down patients’ overall waiting time and improve medical experience satisfaction, which is conducive to alleviating time toxicity in breast cancer patients (15).

On the premise of strictly following standardized treatment cycles determined by tumor pathological indicators and clinical guidelines, clinicians may prioritize regimens with fewer routine follow-up visits for patients with academic or career commitments. Flexible appointment scheduling and streamlined pre-treatment examination workflows can also be adopted to relieve conflicts between fixed therapeutic schedules and participants’ study or work arrangements. Eligible patients can also receive home-based services to cut commuting time. Home-based services may be implemented for eligible patients. In addition, adverse reaction monitoring via smart healthcare platforms may reduce unplanned readmissions and associated time consumption.

### Promoting shared decision-making and precise information support

This study identified doctor-patient information asymmetry, constrained patient voice during screening, and insufficient information support and decision-making pressure during treatment preparation as core drivers of time toxicity. Many patients reported negative experiences due to inadequate information provision. During screening, the absence of timely updates during waiting periods subjectively prolonged perceived wait times and triggered severe anxiety. During treatment preparation, highly technical communication and limited patient comprehension meant that patients’ time-related concerns were rarely addressed.

Gupta et al. (16) noted that cancer patients consider both survival benefits and hidden treatment costs. However, clinicians often lack quantitative data on time toxicity, leading to insufficient decision support and potential bias. Time toxicity also varies substantially across treatment modalities; chemotherapy and radiotherapy involve more frequent visits and greater time expenditure than targeted, immune, or endocrine therapies (14). Therefore, integrating time toxicity into breast cancer treatment decision-making is essential to help patients understand the time costs and benefits of different options and alleviate decision-making pressure.

We only regard the phased, precise information support model as an exploratory direction for subsequent interventional research, derived from our core thematic findings of pervasive doctor-patient information asymmetry and decisional anxiety. During screening and diagnosis, clinics may pilot simplified popular science videos and offline thematic lectures to explain medical workflows, fill information gaps and alleviate waiting-induced anxiety. During treatment preparation, physicians may collaboratively formulate personalized regimens to help patients establish realistic expectations of time investment (17). Clinicians should avoid imposing tight deadlines for treatment plan confirmation, as compressed decision windows coupled with information asymmetry jointly exacerbate patients’ time burden and decisional anxiety; adequate consultation time alongside standardized preoperative educational materials can mitigate this specific form of time toxicity. During treatment implementation, comprehensive care information can be disseminated via online and offline platforms, with specialized breast nurses readily addressing inquiries about catheter maintenance, side-effect management, and rehabilitation training to enhance information transparency and reduce cognitive bias (18). Implementing a collaborative care model to reduce the opportunity cost of time toxicity.

This study confirmed that interrupted development, barriers to social reintegration, and role-related time conflicts during treatment contributed to substantial opportunity costs associated with time toxicity, consistent with the findings of Quinn et al. (19). Most participants held multiple roles related to work, education, and family. Frequent hospital visits and hidden time costs forced many to suspend studies, resign, or take extended leave, disrupting plans for graduation, employment, and promotion. After treatment, some patients encountered obstacles to social reintegration due to physical limitations and changes in appearance, requiring time to adapt to daily life (20). In addition, time conflicts during role transition and life adjustment in the treatment preparation phase imposed additional pressure as patients rapidly reorganized work and family responsibilities.

Accordingly, digital health tools may support remote symptom monitoring, real-time feedback, and personalized guidance. Some routine follow-ups may be completed at home to reduce unnecessary hospital visits and waiting time, preserving more time for role functioning and personal development. Primary care providers may offer home visits for rehabilitation guidance, psychological support, and catheter maintenance during treatment intervals to reduce travel burdens.

To address social alienation and emotional distress, a peer support platform may be established to facilitate experience sharing and mutual connection. Psychological nurses and specialized nurses may jointly provide tailored counseling for role adjustment and social reintegration to relieve guilt and anxiety and shorten adaptation periods. At the policy and social support level, improved social welfare and vocational support may help patients return to work or school and reduce opportunity costs stemming from disrupted life trajectories.

### Limitations

One obvious limitation is that radiotherapy patients were not recruited, which prevents comprehensive comparison of time toxicity across all breast cancer treatment modalities. Our hospital lacks radiotherapy equipment, so all participants only received systemic therapy during interviews and needed to complete radiotherapy at external medical institutions. Future multi-center studies incorporating radiotherapy patients can supplement relevant comparative data.

In addition, this study only explored time toxicity in the active treatment phase and excluded the rehabilitation period. The single-center design reduces the generalizability of our results. We failed to quantify time costs induced by postoperative recovery and treatment-related complications, which deserves further investigation. Multi-center research is needed to formulate generalizable intervention strategies for young and middle-aged breast cancer patients.

Third, several sampling constraints should be noted. Only literate patients with unimpaired cognition were included, so our results cannot be extrapolated to patients with low literacy. All participants were recruited from inpatient wards, excluding those receiving community outpatient or home-based care. Furthermore, patients receiving palliative or end-of-life care were not enrolled. Their distinctive time burdens are absent from our dataset, restricting full understanding of time toxicity throughout the entire disease trajectory. Future research shall broaden sampling scope to include these understudied populations.

## Conclusion

Using a journey mapping approach and in-depth interviews, this study identified dynamic changes in time toxicity among young and middle-aged breast cancer patients across four dimensions. Core characteristics included passive waiting and information anxiety during screening, superimposed explicit and implicit time costs during treatment, routinized long-term care, and periodic follow-up anxiety. These findings provide a framework for developing targeted, phase-specific interventions to address time toxicity in clinical practice.

## Data Availability

The raw data supporting the conclusions of this article will be made available by the authors, without undue reservation.
